# Supplementary material for: Fatty Acid ABCG Transporter GhSTR1 Mediates Resistance to Verticillium dahliae and Fusarium oxysporum in Cotton
Source: Plants (Basel). 2025 Feb 5;14(3):465. doi: 10.3390/plants14030465 (PMC11820032; doi:10.3390/plants14030465)
Supplement: Supplementary file 1 [file plants-14-00465-s001.zip › Supplementary Materials Table S1.pdf]

Table S1. Primers used in this study.

Primer name	Sequence (5′–3′)	Purpose
q-*GhUBQ7*-F	GAAGGCATTCCACCTGACCAAC	qRT-PCR
q-*GhUBQ7*-R	CTTGACCTTCTTCTTCTTGTGCTTG	
q-*Atactin2*-F	GCACCCTGTTCTTCTTACCG	
q-*Atactin2*-R	AACCCTCGTAGATTGGCACA	
q-*GhSTR1*-F	GGCATCGACATCATTCATAAACC	
q-*GhSTR1*-R	CACCTTCTCCACCACACTATAAG	
q-*AtSTR1*-F	CGACTTAGACAAACTTCTCGG	
q-*AtSTR1*-R	CGGCGTATGTTATGCAAGAC	
GhSTR1-F	ATGGCGAAGACAGGGCG	Clone of *GhSTR1*
GhSTR1-R	CATGGGACAAAAATCCCAG	
CM-*GhSTR1*-F	GAATTCTGACATGGAAGAAGTGTTGGAC	Clone of *GhSTR1*
CM-*GhSTR1*-R	GGTACCTAATGGCGGCGAATGTGAAG	silencing fragments
SALK_129014-LP	CCCACACGAACGTTAAATTTC	holomorphic
SALK_129014--RP	GACCGAAGTCGGAGAAGAATC	identification
LBb1-BP	GCGTGGACCGCTTGCTGCAACT	
ITS-F	AAAGTTTTAATGGTTCGCTAAGA	Fungal biomass
VE1-R	CTTGGTCATTTAGAGGAAGTAA	assay
