# Supplementary figures and images for: Fatty Acid ABCG Transporter GhSTR1 Mediates Resistance to Verticillium dahliae and Fusarium oxysporum in Cotton
Source: Plants (Basel). 2025 Feb 5;14(3):465. doi: 10.3390/plants14030465 (PMC11820032; doi:10.3390/plants14030465)

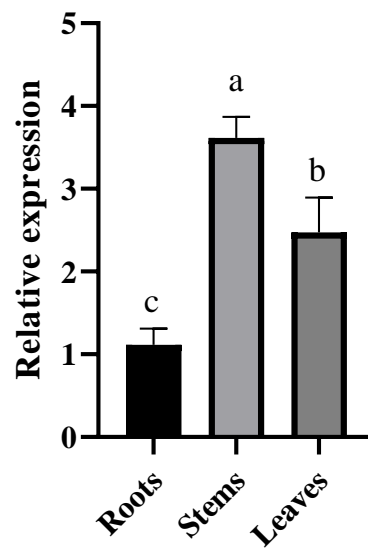

Figure S1. Relative expression of *GhSTR1* in cotton roots, stems, and leaves as measured by qRT-PCR.

Supplement: Supplementary file 1 [file plants-14-00465-s001.zip › Supplementary Materials Figure S1.pdf]
